# Biology and Genomics of Viruses Within the Genus *Gammabaculovirus*

**DOI:** 10.3390/v3112214

**Published:** 2011-11-10

**Authors:** Basil Arif, Shannon Escasa, Lillian Pavlik

**Affiliations:** Laboratory for Molecular Virology, Great Lakes Forestry Centre, Sault Ste. Marie, Ontario P6A 2E5, Canada; E-Mails: sescasa@nrcan.gc.ca (S.E.); lipavlik@nrcan.gc.ca (L.P.)

**Keywords:** Hymenopteran baculoviruses, biocontrol agents, genomics

## Abstract

*Hymenoptera* is a very large and ancient insect order encompassing bees, wasps, ants and sawflies. Fossil records indicate that they existed over 200 million years ago and about 100 million years before the appearance of *Lepidoptera*. Sawflies have been major pests in many parts of the world and some have caused serious forest defoliation in North America. All baculoviruses isolated from sawflies are of the single nucleocapsids phenotype and appear to replicate in midgut cells only. This group of viruses has been shown to be excellent pest control agents and three have been registered in Canada and Britain for this purpose. Sawfly baculoviruses contain the smallest genome of all baculoviruses sequenced so far. Gene orders among sequenced sawfly baculoviruses are co-linear but this is not shared with the genomes of lepidopteran baculoviruses. One distinguishing feature among all sequenced sawfly viruses is the lack of a gene encoding a membrane fusion protein, which brought into question the role of the budded virus phenotype in *Gammabaculovirus* biology.

## 1. Introduction

Baculoviruses have been recently been reclassified into four genera to accommodate viruses that could not fit into the previous classification scheme. The present classification is as follows:

Genus *Alphabaculovirus*—nucleopolyhedroviruses (NPVs) of *Lepidoptera.*
Genus *Betabaculovirus*—Granuloviruses (GVs) of *Lepidoptera.*
Genus *Gammabaculovirus*—NPVs of *Hymenoptera*.
Genus *Deltabaculovirus*—NPVs of *Diptera*.

Fossil records from the upper Triassic and Jurassic era indicated that insects from the order *Hymenoptera* have existed on earth for at least 200 million years. Sawflies, which are related to bees and wasps, derive their name from the manner in which females lay their eggs on tree branches to give a saw-like appearance. They are defoliating insects and have been forest pests in many parts of the world with different species preferring to forage on different trees or groups of trees.

Baculoviruses infect at least 20 different species of sawflies [1] and some have been shown to be excellent forest insect pest control agents. Molecular analysis and phylogeny of the polyhedrin gene suggested that GVs (*Betabaculovirus*) separated from lepidopteran NPVs (*Alphabaculovirus*) later than these two from baculoviruses of *Hymenoptera* (*Gammabaculovirus*) [2,3]. It is now believed that baculoviruses have undergone host-dependent evolution during their development.

Earlier trials to control sawflies relied on the use of chemical insecticides to manage infestation. Calcium arsenate was sprayed on infested plantations in the province of Quebec in 1941. DDT (**d**ichloro**d**iphenyl**t**richloroethane) was also used and both chemicals proved effective in protecting 3–10 year old plantations. The NPV of the red-headed pine sawfly, *Neodiprion lecontei* (NeleNPV), was isolated from cadavers in 1950 and, immediately the question was raised on its potential to control serious infestations of pine plantations [4]. This was particularly timely since about a decade before that, the European spruce sawfly was controlled quite efficiently by its native virus, *Gilpinia hercyniae* NPV (GiheNPV) [5]. Shortly thereafter, trials with NeleNPV proved its efficacy as a pest control agent [4]. In the late 1930s and the early 1940s, the European spruce sawfly was a serious pest in the eastern parts of Canada and the virus that was introduced with mist blowers by Balch and Bird caused an effective collapse of the infestations within two years [5].

In Europe and to a lesser extent in North America, the European pine sawfly, *Neodiprion sertifer*, is a pest of various growth stages of pines. Bird [6] imported an NPV from Sweden to Canada and used it to effectively control infestation in the eastern part of the country. A similar, or possibly the same, NPV was effective in controlling infestations in various parts of Europe and in the former Soviet Union [7]. The red-headed pine sawfly is a pest of jack pine and red pine plantations in North America. Cunningham *et al*. [8] demonstrated excellent control of infestations with NeleNPV.

Early studies by Bird [9] and Bird and Whalen [10] on the replication of *G. hercyniae* and *Neodiprion americanus banksianae* NPVs showed that the viruses replicated in two stages. The investigators termed the first stage as the “free state” of the virus. The second stage was referred to as the virus in “growing polyhedra”, which is the occlusion process of the “free state” virus that now we refer to as the occlusion derived virus (ODV). Bird [9] noted that the virus replicated in midgut cells only and mortality occurred at 4–7 days post infection [11]. To date, there is no report describing the transmission of virus from an infected cell to a healthy one, that raises the question whether infection is confined to primary target cells or some mechanism exists that allows progeny virus to exit cells and infect healthy ones.

This manuscript will cover some historical perspective to illustrate the seriousness of the sawfly pest problem in the past, the biology of the virus and its interaction with the larval host and finally some genomics of sequenced hymenopteran viruses with emphasis on genome peculiarities in comparison to the genomes of lepidopteran baculoviruses.

## 2. Results and Discussion

### 2.1. Biology and Virus Host Interactions

Over the years, hymenopteran baculoviruses have shown to be excellent control agents of their larval hosts, which stems to a very large extent from the habitat of the insect as well as the strategy of virus replication. Sawflies are gregarious insects that gather as “colonies” on trees. Members of each colony are in very close proximity and contact with other cohorts. It has been known for a long time that the virus causes sloughing of infected cells and causes what has been termed infectious diarrhea [11] which leads to rapid dissemination of the virus in the insect colony. This is an excellent example of a virus spread in a high density population. Indeed, the introduction of one infected insect into a healthy colony leads to rapid collapse of the colony from virus disease (previously communicated by the late John Cunningham). The successes of hymenopteran baculoviruses in the control of their larval host led to their registration as pest control agents for large-scale use. To date, three hymenopteran baculoviruses have been registered for use in the field against their natural hosts. They are the NeleNPV (Lecontvirus), the Neodiprion sertifer NPV (NeseNPV) (Neocheck-S) and Neodiprion abietis NPV (NeabNPV) (Abietiv). Registration requirements vary from one country to another but generally require viruses undergo extensive field efficacy test, safety evaluation on non-target organisms and molecular characterizations. Certainly, a total sequence of a viral genome appears to facilitate the registration process with regulatory agencies. Indeed, it has become relatively easy and inexpensive to sequence a viral genome and regulatory agencies appear to prefer having a published complete sequence.

Studies on different hymenopteran baculoviruses confirmed earlier reports that they replicate exclusively in midgut cells [9–12]. To date, there is no electron microscopic evidence showing that progeny virus buds out of an infected cell to initiate infection in other cells.

The balsam spruce sawfly, *N. abietis*, has been a cyclical pest lasting 3–4 years in eastern Canada. More recently, outbreaks appear to last for a long time and the present one has been defoliating balsam fir, black spruce and white spruce since 1997 [13]. The previous outbreaks collapsed naturally and it appears that the NeabNPV contributed to the mortality of the collapsed infestation [14]. The potential use of NeabNPV on operational basis to control infestation by the balsam spruce sawfly was studied in detail by Olofsson [15] and Moreau and Lucarotti [12]. Both groups concluded that this virus should prove to be effective in pest management. Indeed, the latter investigators applied the virus aerially in a molasses formulation and concluded that 10^9^ occlusion bodies (OBs) per hectare eventually resulted in 90% collapse of the infestation.

It seems that sawfly viruses are good candidates for creating an epizootic in the insect host. Bird [16] discussed an epizootic by a Gilpinia hercyniae NPV (GiheNPV) introduced into an infestation in 1938 in eastern Canada and the fact that an epizootic caused a collapse of the pest population by 1942. He went on to show that a single application of virus on 40 hectares of Scots pines infested with the European spruce sawfly, control was achieved over three years by a virus epizootic. A similar situation was also reported by Bird and Burk [17] regarding an outbreak of the same insect in Ontario, Canada. A study in Britain demonstrated that a small epicenter of infection of the European spruce sawfly by its native virus did not spread much in the first year but disseminated well through the population in subsequent years [18].

### 2.2. Genomics of Hymenopteran Baculoviruses

As indicated, sawflies belong to an ancient order of insects and fossil records show that they have been on earth since the Triassic period of approximately 200–250 million years ago. *Lepidoptera* are relatively more recent that appeared first in the Cretaceous era of approximately 65–145 million years ago [19,20]. Sequencing of hymenopteran baculoviruses was spawned by the idea that *Hymenoptera* were likely to have harbored baculoviruses much earlier than *Lepidoptera* and that the hymenopteran baculoviruses might be more ancient than those of *Lepidoptera* and could possibly be ancestral viruses. To date, the complete genome sequences of three hymenopteran baculoviruses have been reported. The first two were those of NeleNPV and NeseNPV [21,22]. Two years later the sequence of the NeabNPV genome was reported [23].

Hymenopteran baculoviruses contain the smallest genomes of all sequenced baculoviruses so far. NeleNPV genome is the smallest composed of 81,755 bp, NeabNPV of 84,264 bp and NeseNPV of 86,462 bp [21–23]. The genomes also revealed low G+C residues ranging from 33.3% to 34%. By comparison, the genomes of lepidopteran baculoviruses range from 99,675 bp for *Adoxophyes orana* granulovirus (AdorGV) to 178,733 bp for *Xestia c-nigrum* GV [24,25].

### 2.3. Genome Peculiarities

#### 2.3.1. Repeated Sequences

Not all the three sequenced hymenopteran baculovirus genomes have repeated sequences that are similar to those found in lepidopteran baculoviruses. In the latter group, homologous repeated sequences (*hr’s*) have been found to be dispersed throughout the genome and have been implicated as possible origins of DNA replication and as enhancers of gene expression. *Hr’s* have several tandem repeats of perfect or imperfect palindromes within direct repeats. The direct repeated regions (*dr’s*) in the NeleNPV genome are composed of 2–3 copies of direct repeats of 29–160 bp separated by a stretch 29–439 bp [21]. Six NeleNPV ORFs had internal repeats but none of these ORFs showed any homology to the lepidopteran baculovirus multigene family called baculovirus repeated ORFs (*bro’s*). Interestingly, the NeseNPV genome contains both *hr’s* that are similar to those in lepidopteran baculoviruses as well as *dr’s* found in NeleNPV [22,26]. The presence of *hr’s* in NeseNPV accounts partially for the slightly larger genome. The NeabNPV genome contains two repeat regions each of which have a group of repeat elements with a common imperfect sequence. This sequence is homologous to one of the *dr’s* in the NeseNPV genome [23].

#### 2.3.2. Non-Syntenic Region (NSR)

**Figure 1.**
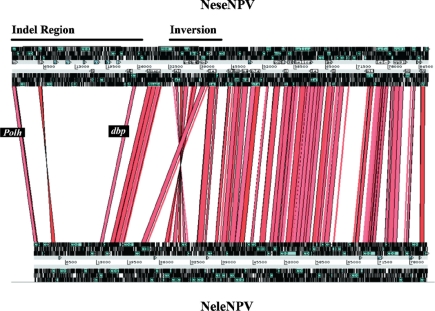
Co-linearity between the NeseNPV and NeleNPV genomes. The two circular genomes are presented in a linear form with the gene encoding polyhedrin (*polh*) placed as ORF 1 in accordance with the convention of linearized baculovirus genomes. Note a major inversion near the middle of the linearized genome and the non-syntenic Indel region between *polh* and *dbp*.

The two genomes of NeseNPV and NeleNPV were remarkably co-linear in terms of gene organization and order except for two regions. There was a major inversion of gene order close to the centre of the linearized genome maps as well as the presence of a very large non-syntenic region between the ORFs encoding polyhedrin (*polh*) and DNA binding protein (*dbp*) (Figure 1). Due to the lack of synteny and close similarity in this region in the two genomes, it has been termed non-syntenic region (NSR) and has been described as a region of insertions and deletions (Indel) [26]. In this region, there are 8 ORF in NeleNPV not found in NeseNPV and 15 ORFs in NeseNPV not found in NeleNPV. A similar region is also recognized in the NeabNPV genome but was not as well characterized [23]. The only two identified ORFs in the NSR with baculovirus homologues encode *mtase1* (equivalent to ac69) and *iap*. Interestingly, the closest matches to these ORFs were found in insects, which gives strength to the previous theory that iap genes might have been derived from the host itself. The *mtase1* ORF is present in the NeseNPV genome and is absent from NeleNPV and NeabNPV [26]. Two ORFs (nele7/nese6) encode a trypsin-like serine protease, which was not found in NeabNPV. The putative protein encoded by nele7/nese6 will be discussed later.

Lauzon and colleagues [26] theorized that the NSR might have arisen by transfer of a cluster of genes and ORFs from an insect host(s) and only those ORFs that afforded the virus a selective advantage in nature were maintained.

#### 2.3.3. Serine Protease

A trypsin-like serine protease has been reported in the NeleNPV and NeseNPV genomes with BLAST matches to insect serine proteases. This putative enzyme has not been reported in other baculovirus genomes. Phylogenetic analysis supported the theory that the ORF encoding this enzyme was acquired by an ancestral hymenopteran baculovirus from an insect host [21,22]. Structural modeling of the enzyme with bovine trypsin showed that the positioning of the catalytic triad of His, Asp and Ser was preserved indicating a likelihood of a functional serine protease [26].

#### 2.3.4. Lack of Homologs to a Membrane Fusion Protein (MFP) and to Immediate Early Protein-1 (IE-1)

A glaring absence from all three sequenced hymenopteran baculoviruses is that of a membrane fusion protein homologous to an F-protein or GP64. The former is found in all group II alphabaculoviruses and in some group I alphabaculoviruses as well as in *Culix nigripalpus* NPV (CuniNPV, genus *Deltabaculovirus*) while GP64 is in group I NPVs [21–23]. Exhaustive searched of the three genomes did not reveal a potential MFP with all the required feature of a transmembrane domain, a signal peptide, conserved serine residues and a furin cleavage site [27,28]. The MFPs mediate the fusion of budded viruses with cell membrane to initiate infection in a susceptible cell. It has been mentioned that hymenopteran baculoviruses are restricted to the midgut and, indeed it is not known if they are released from an infected midgut cell to infect other midgut cells. This replication strategy may preclude the need of a budded virus phenotype, which may have arisen at a later evolutionary event(s) [21–23].

Searches of all three sequenced hymenopteran baculoviruses revealed a conspicuous absence of an ORF encoding an IE-1. This is a pivotal protein that has been shown to be transcribed throughout the replication cycle of lepidopteran baculoviruses. It transactivates many genes expressed early and late in the in the infection cycle [29]. It is possible that another gene may assume this function in hymenopteran baculoviruses but the lack of tissue culture system that supports replication makes it difficult to search for such a function.

#### 2.3.5. Phosphotransferase

Phosphotransferases are proteins involved in tRNA splicing. Genomic analyses showed that NeleNPV, NeseNPV and NeabNPV have homologs to RNA 2′-phosphotransferase KptA/TPT. This family of enzymes is present in eukaryotes and in a very few prokaryotes but have not been previously reported in viruses [30]. The protein in *D. melanogaster* had strong amino acid identity with its homologs in the NeleNPV and NeseNPV genomes [26]. However, the functionality of the enzyme in hymenopteran baculoviruses has not been investigated.

#### 2.3.6. Homologs to Densovirus Capsid Protein

All three sequenced hymenopteran baculoviruses reported the presence of a homolog to an ORF potentially encoding a densovirus capsid protein of unclear functionality in these viruses. All homologs show strong BLAST matches to the capsid protein *Bombyx mori* densovirus [26]. The latter virus replicates in midgut epithelial cells while other densoviruses infect a variety of tissues [31].

#### 2.3.7. Regulator of Chromosome Condensation Proteins (RCC1)

RCC1 proteins play an important role in regulating chromosome condensation by binding to DNA or to proteins associated with chromatin. NeleNPV and NeseNPV have three RCC1 homologs while NeabNPV has two homologs. These homologs have BLAST matches to a number of RCC1 proteins including those from *D. melanogaster* and *Anopheles gambiae* [21–23].

### 2.4. Transcription

To date, there is not tissue culture system that supports the replication of a hymenopteran baculovirus. Since replication in tissue culture cells depends on the production of a budded virus phenotype, it is possible that the lack of a MFP precludes the ability of these viruses to replicate *in vitro*. Limited investigation on DNA replication and gene expression of NeabNPV in the natural host was undertaken [32]. The investigators showed that viral DNA appeared to increase at a fast rate for 30 min. post infections of larvae with 10^4^ occlusion bodies/larva and at a lower rate for the next 70 hours post infection (hpi). By using RT-PCR, they showed that the late expression factor genes, *lef-1*, *lef-2* and DNA polymerase gene (*dnapol*) were transcribed as early as 2 hpi. Transcription of *lef-8* was detected as early as 1 hpi while that of *lef-9* at 6 hpi. These two genes encode subunits of RNA polymerase. Transcription of *gp41* and *p74* was observed at 6 hpi while that of *vlf-1* was detected late at 12–72 hpi. Similarly, *polh* was transcribed at 24 hpi. These data show that the temporal control of gene expression in hymentopteran baculoviruses is quite similar to that of the lepidopteran viruses and that this control may be universal in all baculoviruses [32].

## 3. Conclusions

Hymenopteran baculoviruses appear to be more ancient than those infecting *Lepidoptera*. Indeed, phylogenetic studies have shown that the sawfly baculoviruses have radiated before the separation of alphabaculoviruses and betabaculoviruses [21,22,32]. It is understandable that they do not contain a membrane fusion protein because their replication is restricted to the midgut. However, the lack of IE-1 is a bit perplexing and it is likely that some other protein assumes its function in sawfly virus replication.

To date, there is no evidence of how, or even if, progeny virus spreads from one midgut cell to another. This awaits detailed electron microscopic studies to further elucidate the replication cycle of hymenopteran baculoviruses.
